# How Next-Generation Sequencing Has Aided Our Understanding of the Sequence Composition and Origin of B Chromosomes

**DOI:** 10.3390/genes8110294

**Published:** 2017-10-25

**Authors:** Alevtina Ruban, Thomas Schmutzer, Uwe Scholz, Andreas Houben

**Affiliations:** Leibniz Institute of Plant Genetics and Crop Plant Research Gatersleben, 06466 Seeland, Germany; ruban@ipk-gatersleben.de (A.R.); schmutzr@ipk-gatersleben.de (T.S.); scholz@ipk-gatersleben.de (U.S.)

**Keywords:** B chromosome, supernumerary chromosome, evolution, next generation sequencing

## Abstract

Accessory, supernumerary, or—most simply—B chromosomes, are found in many eukaryotic karyotypes. These small chromosomes do not follow the usual pattern of segregation, but rather are transmitted in a higher than expected frequency. As increasingly being demonstrated by next-generation sequencing (NGS), their structure comprises fragments of standard (A) chromosomes, although in some plant species, their sequence also includes contributions from organellar genomes. Transcriptomic analyses of various animal and plant species have revealed that, contrary to what used to be the common belief, some of the B chromosome DNA is protein-encoding. This review summarizes the progress in understanding B chromosome biology enabled by the application of next-generation sequencing technology and state-of-the-art bioinformatics. In particular, a contrast is drawn between a direct sequencing approach and a strategy based on a comparative genomics as alternative routes that can be taken towards the identification of B chromosome sequences.

## 1. Recent Discoveries Related to the Origin and Evolution of B Chromosomes

The origin and evolution of the B chromosomes, which appear to make a non-essential contribution to the overall genome, have puzzled cytogeneticists for over a century. These generally smaller than standard (A) chromosomes are transmitted in a higher than expected frequency, leading to a rise in their number from one generation to the next, in a process termed “drive” [1,2]. The quantum improvement in DNA sequencing power achieved by so-called next-generation sequencing (NGS), along with associated analytical methodologies, now allows for a rigorous investigation of the nucleotide composition of the B chromosomes. The result will finally provide an unequivocal answer to whether or not they harbor genes, whether they affect the function of the genome and how they originated. A number of B-chromosome-carrying species, representing a broad range of taxa, have been targeted in recent years to address these issues. The outcome of applying NGS and extensive cytogenetic analyses has been that the B chromosomes, despite their being non-essential, have been shown to share much in common with A chromosomes, and that they evolved in the various taxa in comparable ways.

Among the plant B chromosome carriers, the major focus on the sequence composition of B chromosomes has been on rye (*Secale cereale*). Based on molecular clock calculations, it has been estimated that rye B chromosomes originated approximately 1.1–1.3 million years ago, 0.4–0.6 million years after the formation of the *Secale* genus [3]. Analysis of flow-sorted B chromosomes has shown that they harbor a substantial amount of A-chromosome-derived DNA sequences. On the basis of these sequences, the B chromosomes represent a multichromosomal mosaic, with the two A chromosomes 3R and 7R making the largest contribution. The distribution of repetitive DNA along the B chromosome is largely similar to that found in the A chromosomes, although certain transposable elements are either noticeably rarer or noticeably more abundant in the B chromosomes than in the A chromosomes. Two repetitive sequences, arranged as tandem repeats, were shown some time ago to be B-chromosome-specific, but NGS has now uncovered a further nine sequences that appear to be strongly enriched in the B chromosomes—these are most likely tandemly arranged, and are concentrated either in the non-disjunction control region or in the pericentromere. Some of B-specific repeats are transcribed in a tissue-type specific manner [4]. Other sequences have clearly been derived from organellar (plastids and mitochondria) genomes, which is similarly the case for the B chromosomes of the grass species (and wheat progenitor) *Aegilops speltoides* [5]. As a result, it has been proposed that the rye B chromosomes arose in a stepwise manner, possibly as a by-product of evolutionary rearrangements of the A chromosome complement. The suggestion is that the progenitor of the B chromosome arose in conjunction with a segmental or whole genome duplication event, during which segments of several A chromosomes coalesced. An independent mode of evolution of the B chromosomes requires that they no longer are able to associate meiotically with their A chromosome progenitor(s). The prediction flowing from this scenario is that B chromosomes are more likely to have arisen in taxa which have experienced major karyotypic rearrangements [3]. The B chromosomes found in wild and cultivated rye populations of diverse geographical origin are structurally highly conserved, an observation which has been taken to suggest that, despite their rapid initial evolution, once they had become established, their rate of further structural change and their accumulation of repetitive sequence became greatly attenuated. Intriguingly, however, the level of nucleotide polymorphism appears to be much higher in B chromosome genic sequences than in their A chromosome homologs [6]. Some of these genic sequences are actively transcribed and their transcripts may well be functional [7]: for example, a copy of *AGO4B* residing on a B chromosome is transcribed and has been shown to possess RNA slicer activity, at least in vitro [8].

Uniquely among the many taxa that carry B chromosomes, only fungal species harbor definitively functional B chromosomes; in some cases, they even endow a selective advantage on the host. Some are known to harbor genes encoding the virulence function, which allows for host colonization, and others genes underlying potentially adaptive traits [9,10]. The single-molecule real-time (SMRT) sequencing of a strain of the fungal pathogen *Fusarium poae* carrying at least one B chromosome has established that the A and B chromosomes differ in their content—specifically, the former harbor few transposable elements and no gene duplications, while up to 25% of the latters’ sequence is composed of transposable elements, and gene duplications are frequent [11]. Similarly, the B chromosome sequence of *Nectria haematococca*, a fungal pathogen belonging to the *Fusarium solani* species complex, comprises a higher proportion of repetitive DNA than does that of the A chromosome complement; its GC content is lower, and it includes both single copy and duplicated genes [12]. The understanding is that fungal B chromosomes represent a part of the genome able to evolve faster than the standard chromosome complement, thereby permitting the rapid development of pathogenicity without disturbing the core genome (see review by Croll and McDonald [13]).

An NGS-enabled comparison of the genomic sequences of 0B and 4B males of the grasshopper species *Eyprepocnemis plorans*, along with the assembled transcriptomes of 0B and 1B females, has revealed ten B chromosome protein-encoding genes, four of which are complete and six truncated [14]. The abundance of transcript derived from half of these genes was significantly higher in the B chromosome carriers, and in some cases, the increase in abundance could be correlated with the number of B chromosome copies present. A gene ontology analysis has suggested that these B-chromosome-encoded genes are predominantly involved in the regulation of cell division, but it has not been established as yet whether the transcripts generated from the B chromosome gene copies are functional.

A sequence analysis of the satellite repeat fraction present in the grasshopper *Eumigus monticola* genome has suggested that one of the autosomes contributed the most for B chromosome formation [15]. Selected satellite repeats and 5S rDNA showed a similar distribution in the proximal third of autosome S8 and the B chromosome. Two repetitive families, which were considered, on the basis of in situ hybridization, to be B-chromosome-specific, were represented by a high copy number. A bioinformatic analysis concluded, however, that both were in fact also to be found in the A genome complement, although at a density which was too low to detect cytogenetically. The observation was taken as supportive of the intraspecific origin of the B chromosomes. Such conclusions remain provisional however, given the dynamic behavior of satellite repeats. Ruiz-Ruano et al. [16] have also analyzed the repetitive DNA content of micro-dissected B chromosomes carried by the migratory locust *Locusta migratoria*, and demonstrated a substantial difference between the proportions of the B and A chromosome sequence represented by repetitive DNA—respectively, 94.9% and 64.1%. Six different satellite repeats were located on the B chromosome, whereas only one member of the A chromosome complement harbored all of these satellite sequences. On this basis, this chromosome, along with a second autosome, which shares histone gene sequences with the B chromosome, have been proposed as the putative donors of the B chromosome sequences. A further feature of this B chromosome is a 17 Kbp segment composed of 29 distinct transposable elements, indicative of the occurrence of multiple insertion events within this region.

The most drastic impact of B chromosomes documented in the literature relates to the jewel wasp (*Nasonia vitripennis*), in which the B chromosomes (also referred to as the “paternal sex ratio” (PSR) chromosomes) are transmitted exclusively via the sperm, and act to eliminate one set of A chromosomes during the zygote’s first mitosis [17,18]. As a result, a female zygote is converted into a male embryo (see review by Aldrich and Ferree [19]). The PSR-induced elimination of the paternal A chromosomes is regulated by post-translational modifications to the histones associated with the sperm’s chromatin [20]. A transcriptomic analysis of the *N. vitripennis* testis has identified a number of PSR-specific transcripts, which may either encode a functional protein or may represent long non-coding RNA [17]. As yet it remains unclear both how these transcripts relate to the key chromatin modifications and what the nature of the controlling mechanism may be.

A plausible example of the de novo formation of a B chromosome has recently come to light in *Drosophila melanogaster*. Although the presence of B chromosomes has been documented in the *Drosophila* genus since at least 1980, they were first noted in *D. melanogaster* karyotype as recently as 2014 in an established stock containing the *mtrm^126^* allele of the *matrimony* (*mtrm*) gene. Importantly, no B chromosomes had been identified either in the stock from which the *mtrm^126^* mutant line was created, or in stocks bearing different *mtrm* alleles [21]. The implication was that a B chromosome had formed over the course of the ten-year period of the stock’s maintenance. These particular B chromosomes were highly heterochromatic and resembled chromosome 4 with respect to the arrangement of certain heterochromatin-related satellite repeats. Their presence has been associated with the meiotic non-disjunction of achiasmate copies of chromosome 4 in females. The B chromosomes do not apparently possess a strong drive mechanism and are thought to be mitotically unstable [21]. While these de novo formed B chromosomes appear to offer an appropriate model for revealing the origin and evolution of B chromosomes, as yet little information has been gathered concerning their sequence composition.

The acquisition of genomic sequence obtained from individual cichlid fish (*Astatotilapia latifasciata*) either carrying or not carrying B chromosomes, as well as that of micro-dissected B chromosome sequence has permitted some clarification regarding the origin and evolution of the B chromosomes present in this species [22]. The sequence data have suggested that a proto B chromosome formed before the diversification of the main lineages of the Lake Victoria population, induced by segmental duplications occurring within the autosomes. Three different A chromosomes appear to have provided most of the material making up the B chromosome, but there remains a level of similarity to most of the A chromosomes. The development of a proto B chromosome appears to reflect an accumulation of A chromosome-derived fragments, followed by a burst of sequence amplification and the establishment of a drive mechanism. Besides the large proportion of repetitive DNA present on the B chromosomes (larger than that on the A chromosomes), a number of genic sequences are also present, although most of these are gene fragments. The few genes remaining intact present are likely either to have been transferred rather recently to the B chromosomes, or to possess functions important for the maintenance and transmission of the B chromosomes. Among the genic sequences detected which have retained a similar structure to their A chromosome homologs, several are associated with the process of cell division, namely the structure of the kinetochore, recombination, cell cycle progression and microtubule organization. Valente et al. [22] have suggested that those that appear to be transcribed are likely involved in the transmission of the B chromosomes. The PCR amplification of some B chromosome sequences present in the cichlid fish *Metriaclima zebra* and six other Lake Malawi cichlids has revealed a link between the B chromosomes and the sex of the zygote [23], which has been reported to be the case as well for a number of species [24]. The underlying mechanism of this association is either an effect of drive, which results in one of the sexes carrying a higher number of B chromosomes, or represents the outcome of a mechanism that ensures that B chromosomes are more frequent in males or females in which it drives [23].

A high-quality assembly of the domestic dog genome sequence was combined with a comparative cytogenetics approach by Becker et al. [25] to address aspects of genome architecture in a selection of canid species. As part of the study, the sequence composition of the B chromosomes of the common fox (*Vulpes vulpes*) and two subspecies of raccoon dog (*Nyctereutes procyonoides*) was revealed in some detail. In addition to the identification of additional copies of the proto-oncogene *cKIT* [26], the presence of other genomic regions shared by A and B chromosomes was successfully demonstrated. The inference was that the canid B chromosomes likely arose in a single ancestral species as a byproduct of a genome rearrangement event(s), which led to speciation in the Canidae. The canid B chromosomes represent a pool of duplicated sequences, including cancer-associated genes, many of which are associated with chromosome breakpoints [25,27].

The Siberian roe deer (*Capreolus pygargus*) and grey brocket (*Mazama gouazoubira*) are both B-chromosome-carrying members of the Cetartiodactyla [28]. In contrast to the evolution of the canid B chromosomes, in this case, the B chromosomes seem likely to have originated independently. The acquisition of sequence from flow-sorted B chromosomes has enabled a demonstration that both harbor mainly repetitive DNA, with some representation of autosomal sequences undergoing pseudogenization and of amplified non-repetitive sequences. However, both the composition of the repetitive DNA and the spectrum of A-chromosome-derived sequences present differed greatly between the two species. Those in *C. pygargus* harbored at least two duplicated A chromosome regions containing three genes, and the level of heterozygosity and the number of haplotypes was high. In contrast, the *M. gouazoubira* B chromosomes were relatively homogeneous. There were 26 duplicated regions, harboring 34 intact and 21 partial gene sequences. The presence of both the proto-oncogenes *cKIT* and *RET* [25] in the *M. gouazoubira* B chromosomes suggests that the A chromosome genomic regions that become involved in B formation are not random [28].

Chromosome-specific probes generated from flow-sorted B chromosomes of the rodent *Holochilus brasiliensis* have been exploited for in situ hybridization in *Oryzomyini* spp. [29]. These experiments have revealed some common sequences, referred to as the “anonymous *Oryzomyini* shared heterochromatic region” (OSHR), which are found in 12 of the 15 species analyzed. The OSHR is thought to have arisen 3.0–7.8 million years ago on the sex chromosomes of an ancestral species, spreading later to some autosomes as well as to established B chromosomes through the action of transposable elements. An independent evolution of B chromosomes in the genus *Oryzomyini* has been proposed by Ventura et al. [29]. As yet, the origin of the heterogeneous B chromosomes remains unclear. The proposition is that, in rodents, both the autosomes and the sex chromosomes have contributed to the evolution of B chromosomes [30]. The involvement of sex chromosomes has also been suggested in the frog species *Leiopelma hochstetteri* [31].

## 2. The Acquisition of Sequences Enriched in B Chromosome

Differences between the A and B chromosomes with respect to their size, structure, and pattern of meiotic pairing behavior offer the opportunity to isolate the B chromosomes via micro-dissection. In plant species where it is difficult to synchronize mitotic division across many cells, advantage can be taken of the natural synchrony associated with meiosis, particularly in the anthers, where large numbers of pollen mother cells passage through meiosis simultaneously. In the earliest reported use of micro-dissection to obtain B chromosome-specific sequences, Sandery et al. [32] attempted to clone into lambda phage DNA obtained from a very large number of rye B chromosomes; although the approach was rather inefficient. The introduction of PCR was responsible for a quantum leap in efficiency, and this technology lies behind most current protocols for chromosome micro-dissection and the subsequent handling of the DNA [33,34,35,36]. Successful in situ painting of B chromosomes (e.g., rye [37], *Brachycome dichromosomatica* [38]) with labelled DNA generated after microdissection was possible because of the enrichment of chromosome-specific repetitive sequences, rather than the chromosome specific low- and single-copy sequences. A list of B chromosomes successfully isolated via micro-dissection is given in Table 1. An alternative route to acquiring chromosome-specific DNA takes advantage of the power of flow-sorting to separate chromosomes on the basis of their size [39]. A major advantage of this approach is that it isolates orders of magnitude higher numbers of chromosomes than is feasible using micro-dissection. The resulting DNA can be amplified using either degenerate oligonucleotide primed PCR [40] or Phi29 multiple displacement amplification [41]. The latter technique is more effective where longer amplicons (5–30 Kbp) are preferred [42]. Species for which flow-sorting has been successfully used to purify B chromosome DNA are listed in Table 2.

## 3. The In Silico-Based Identification of B Chromosome-Enriched Sequences

**Figure 1:**
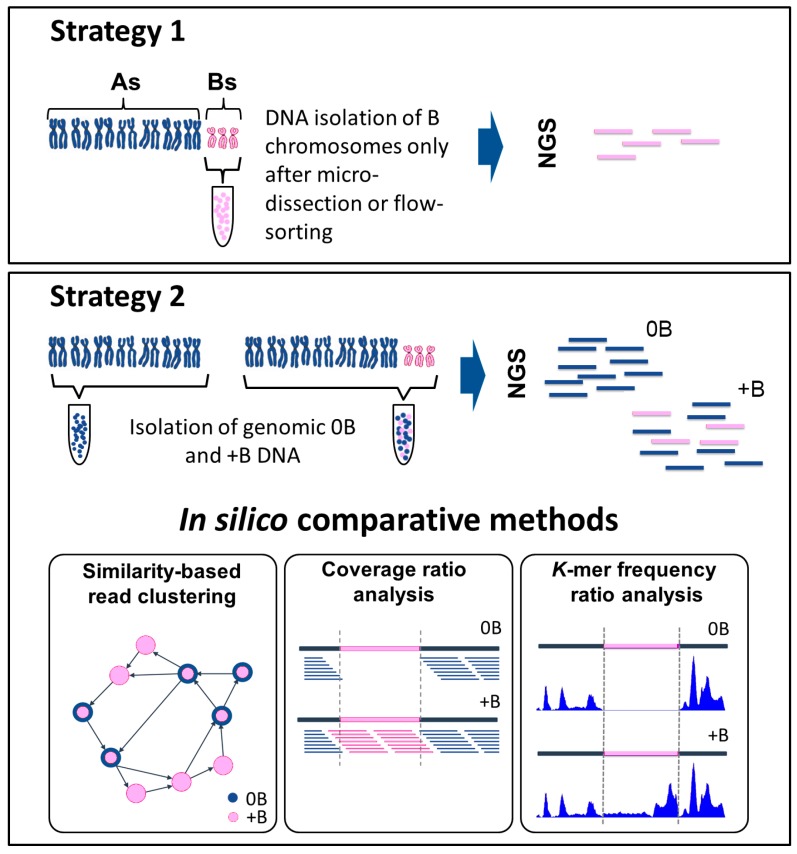
Direct and indirect methods used to identify B chromosome sequences using next generation sequencing (NGS). Strategy 1: the direct method. This approach requires a prior step, in which the B chromosomes are isolated either by micro-dissection or by flow-sorting. Strategy 2: the indirect method. This method requires the acquisition of sequence data from both an individual carrying a B chromosome(s) (+B dataset) and a related individual lacking any B chromosome(s) (0B dataset). The two datasets are compared using three alternative methods. In “similarity-based read clustering”, a graphically based analysis is performed using, for example, the RepeatExplorer pipeline. Sequence information is transformed into graphical structures (vertices correspond to sequence reads and edges characterize the overlap between reads). Differences (presence/absence of sequence reads) in the 0B and +B datasets affect the clusters, and are used to distinguish B chromosome sequences. The two-colored circles indicate reads containing sequences from 0B and +B probes. The “coverage ratio analysis” requires an initial alignment of reads, using an alignment pipeline such as Burrows-Wheeler Alignment tool (BWA). Differences in the read coverage ratio indicate B chromosome-derived candidate regions. The pink section illustrates an example of a putative candidate region, which features the absence of reads in the 0B dataset and their presence in the +B dataset. In the “*k*-mer frequency ratio analysis” approach, a program such as the Kmasker pipeline identifies differences in the *k*-mer frequency ratio. The illustration shows an example of a B chromosome segment (shown in pink) in which the *k*-mer frequency is low or zero in the 0B dataset, but high in the +B dataset. Both the coverage ratio and *k*-mer frequency ratio analyses, but not the similarity-based read clustering approach, require a reference sequence.

Various strategies have been elaborated to identify B chromosome sequences from NGS-acquired data. This section summarizes the differences between the direct and indirect (comparative) methods (Figure 1).

### 3.1. Strategy 1—The Direct Route: Isolating, then Sequencing Micro-Dissected or Flow-Sorted B Chromosomes

Once B chromosomes have been isolated by either micro-dissection (Table 1) or flow-sorting (Table 2), it is possible to derive their nucleotide content by standard DNA sequencing approaches. The benefit of this direct method is that there is an a priori assurance that most of the sequences generated are harbored by a B chromosome complement. Employing sufficient sequencing depth, in conjunction with the deployment of advanced bioinformatic tools such as the “targeted chromosome-based cloning via long-range assembly” method [58] can generate sequence assemblies of high quality. Data acquired from a low sequencing depth experiment cannot produce sufficient sequence coverage to allow for a reliable assembly. The major problem encountered with sequencing DNA from micro-dissected material is the noise generated by contamination from non-target chromosomes, from non-target species and from PCR amplification bias. Thus, sequence reads should always be tested (where possible) against reference genome sequences. Here, high specificity and sequence uniqueness is required to identify B chromosome-specific fragments.

Similar to the micro-dissection approach, flow-sorted chromosomes offer a significant reduction in sample complexity, since a specific chromosome can be purified for sequencing. An effective method of sequencing flow-sorted material platform is the so-called “Chicago Hi-C scaffolding” approach, since it requires only small amounts of template DNA [59]. The ability to assemble long sequence scaffolds aids in assessing co-linearity and synteny between B and A chromosomes, and in addressing the origin of B chromosomes sequences. The major limitation encountered with flow-sorting is the difficulty of discriminating between B and fragmented A chromosomes. Measurable progress has been made in recent years towards minimizing this source of contamination [60].

### 3.2. Strategy 2—The Indirect Route: Comparing Whole Genome Sequence Acquired from Individuals Carrying and Not Carrying B Chromosomes

Inferring a B chromosome location for a given sequence from whole genome sequence data requires a comparison between datasets from a pair of (preferably related) accessions, one of which carries one or more B chromosomes (+B) and the other does not (0B). In principle, the approach identifies peaks where the ratio of aligned sequences is significantly higher in the +B dataset than in the 0B dataset. These regions are identified as putative candidates that are enriched in B chromosome sequences. Here, three different methods have been suggested to identify B chromosome-enriched sequences. The use of several independent +B and 0B identification methods helps to reduce the number of false positives.

#### 3.2.1. Similarity-Based Read Clustering

B chromosome-enriched sequences, such as satellite DNA, retrotransposons, and organelle-derived sequences, can be identified by the similarity-based clustering of NGS reads, as attempted by the RepeatExplorer pipeline, which identifies clusters of frequently overlapping reads, and interprets these as parts of repetitive elements [61]. In addition, the pipeline estimates copy numbers, based on the frequency of duplicate reads. It is able to connect adjacent sequence clusters via the use of paired-end sequence reads. Furthermore, it performs BLAST nucleotide and protein sequence (BLASTN and BLASTX) similarity searches [62] against specialized databases of repetitive elements and repeat-encoded conserved protein domains, which supports the annotation of repetitive elements. To reveal the presence of repetitive elements on a B chromosome, the analysis can be run in a comparative mode, performing a simultaneous clustering of reads from the +B and 0B samples. The structure of the clusters can be investigated using the SeqGrapheR program [61]. The approach has been applied with some success in both rye [3] and *Plantago lagopus* [63].

#### 3.2.2. Coverage Ratio Analysis

The “coverage ratio analysis” can be performed by mapping genomic reads against a reference genome [22], as is cited in the manuscript. However, it could be also performed by mapping genomic reads against a reference transcriptome as performed by Navarro-Dominguez et al. [14]. The method works by aligning the +B and 0B dataset, looking for differences in the sequence read coverage ratio (Figure 1). Alignment software such as Burrows-Wheeler Alignment tool (BWA) [64] and Bowtie [65] can be used to construct sequence alignment/maps [66]. Subsequently, the constructed SAM/BAM files are investigated for regions with different numbers of aligned reads. The B chromosome sequence content of the cichlid fish *A. latifasciata* was determined from high coverage whole genome sequence (acquired with an Illmuina HiSeq platform, San Diego, CA, USA) of individuals with and without the B chromosomes, and the reads were mapped onto a reference genome—in this case, that of the related cichlid species *M. zebra* [22]. The coverage ratio analysis revealed that the B chromosomes contain thousands of sequences which have copies on almost every A chromosome. Although most of the genic sequences on the B chromosomes have been fragmented, a few do appear to be intact. Subsequent sequence analysis of micro-dissected *A. latifasciata* B chromosomes has confirmed this conclusion [22].

#### 3.2.3. *k*-mer Frequency Ratio Analysis

A third possible approach is referred to “*k*-mer frequency ratio analysis.” Here, the critical variable is the *k*-mer frequency ratio (Figure 1). A *k*-mer is defined as a sequence fragment of length *k*. The method relies on the construction of a set of such *k*-mer indices covering all sequence motifs occurring in the dataset. Two programs designed to perform this task are Tallymer [67] and Jellyfish [68]. The Kmasker tool [69] can be applied to run the *k*-mer frequency ratio analysis. In addition to its core functionality of masking repetitive elements and identifying low copy sequences, Kmasker can also be used to design both probes for in situ hybridization [70] and single nucleotide polymorphism markers.

The approach was applied in the carnivorous plant species *Genlisea* to study its divergent genome size evolution [71]. In this regard, when comparing the two-sister species *Genlisea nigrocaulis* and *Genlisea hispidula* in their repeat composition, the approach revealed sequence candidates that were involved in the genome size expansion, which is a similar experiment as comparing 0B and +B datasets.

### 3.3. Benefits and Merits of Indirect and Direct Strategies

The major advantage of the indirect over the direct strategy lies in its not requiring a technical intervention (micro-dissection or flow-sorting), which not only incurs cost, but also introduces an unavoidable degree of contamination by off-target material. While most of the unwanted sequence can be excluded using bioinformatics approaches, this further intervention adds yet another intermediate step. Nevertheless, the direct approach gains from the fact that the bulk of the sequence acquired is relevant, while in the indirect approach, the opposite is the case, since most of the sequence acquired originates from the A chromosome complement or from the organellar genomes. Contamination in the template acquired by micro-dissection is likely to derive from off-target species (microorganisms, human) rather than from the host, whereas for the flow-sorted template, the major source of contaminating DNA is likely to be the host’s A chromosome complement and/or organellar DNA. Where a reference genome sequence has been established, much of the contamination should be identifiable using homology searches, except for sequences that are shared between the B and A chromosomes. This is less obviously the case for a template acquired from micro-dissected chromosomes, as in this case, the source of the contamination is unknown. The challenge for the indirect method is to set an appropriate threshold that minimizes type I error, while still retaining a sufficient number of sequences. Defining this threshold depends on the sequencing depth, the sequence diversity to reference genome sequence and the probability of assembly error. Thus, all sequences identified via the indirect route are associated with a level of uncertainty. In general, the indirect approach is most effective for the discovery of sequences that are abundant on the B chromosomes. In some situations, technical considerations can suggest one method as more suitable than the alternative. For instance, where it is not possible to boost the number of somatic cells undergoing mitosis, flow-sorting becomes inefficient. Similarly, micro-dissection is difficult to carry out where the target chromosome cannot be readily identified on the basis of its morphology. Combining direct and indirect approaches can be an effective strategy, since the outcome of one can be used to validate the outcome of the other.

### 3.4. An In Silico Method Used to Identify B Chromosome Sequences

One way of assigning the origin of specific sequences to the B chromosome is to make use of synteny between closely related species, a phenomenon whereby interspecific gene order is maintained, at least within relatively short genomic segments. The “genome zipper approach” [72], which exploits this conservation of gene order, has been used in a number of plant species to order and structure NGS sequences [3,72]. As demonstrated in rye [3], the “genome zipper approach” can be extended to B chromosome sequences, once candidate sequences have been identified by a BLASTN analysis against an appropriate reference genome sequence.

## 4. Conclusions

Combining NGS with state-of-the-art bioinformatics is providing new ways of identifying sequences specific to B chromosomes, revealing a wealth of molecular data relevant for the study of their origin and evolution. Based on sequence data obtained from animal, plant and fungal B chromosomes, the present consensus is that the B chromosomes are composed of duplicated segments derived from potentially multiple A chromosomes, with the addition of some organellar DNA (see review by Houben et al. [73]). Some B chromosomes contain paralogs of A-chromosome-located genes, either as intact or as degenerate sequences. Genic sequences on the B chromosomes do make some contribution to the host transcriptome [74,75]. B-chromosome-specific repeats tend to be derived from the amplification of A chromosome coding and non-coding sequences [76]. The similarities between the B chromosomes and both the so-called “double minute” chromosomes and homogeneously staining regions has suggested that these structures were formed in a comparable manner [76]. There may be parallels between B chromosomes and marker chromosomes in tumor tissue formed by chromothripsis, a process in which several distinct chromosomal regions simultaneously fragment and subsequently are imperfectly reassembled [77]. Human small supernumerary marker chromosomes may serve as an appropriate model for the early evolution of the B chromosomes [78], although these do not share the drive mechanism characteristic of the B chromosomes. Taking into account the growing number of species for which B chromosome-located genic sequences with possible functions have been reported, B chromosomes cannot be considered as “genetically inert” any more. However, their physiological importance still remains at best sketchily understood.

Modern sequencing and bioinformatics methods can be expected to shed new light on the B chromosomes and thereby improve our knowledge of their genomic dynamics. A detailed understanding of the workings of the (peri)centromere will be needed before the mechanistic basis of their characteristic drive can be unraveled. Further progress in RNA sequencing technology will allow for a more rounded picture of the effect on the transcriptome of the B chromosomes to be generated. Additional analysis of the B chromosomes can be expected to provide exciting information relevant to the rapid genome changes that can occur in higher eukaryotes.

## Figures and Tables

**Table 1:** Isolation of B chromosomes by microdissection.

Species	Reference
Rye (*Secale cereale*)	[32,37,43]
Hawks beard (*Crepis capillaris*)	[44]
*Brachycome dichromosomatica*	[38]
Maize (*Zea mays*)	[45]
Dung beetle (*Dichotomius sericeus*)	[46]
Locust (*Locusta migratoria*)	[16,47]
Grasshopper (*Podisma kanoi*)	[48]
Grasshopper (*Podisma sapporensis*)	[49]
Grasshopper (*Abracris flavolineata*)	[50]
Hylid frog (*Hypsiboas albopunctatus*)	[51]
Fish (*Moenkhausia sanctaefilomenae*)	[52]
Fish (*Astyanax scabripinnis*)	[53]
Cichlid fish (*Astatotilapia latifasciata*)	[22]
Fishes genus *Astyanax*: *A. paranae*, *A. fasciatus*, *A. bockmanni*	[54]
Fish (*Prochilodus lineatus*)	[55]
Pacific giant salamander (*Dicamptodon tenebrosus*)	[56]
Korean field mouse (*Apodemus peninsulae*)	[57]

**Table 2:** Isolation of B chromosomes by flow sorting.

Species	Reference
Rye (*Secale cereale*)	[3,4]
Red fox (*Vulpes vulpes*)	[26]
Oryzomyines (*Holochilus brasiliensis*)	[29]
Siberian roe deer (*Capreolus pygargus*)	[28]
Grey brocket deer (*Mazama gouazoubira*)	[28]
